# The efficacy of posterior wall isolation in atrial fibrillation ablation: A systematic review and meta‐analysis of randomized controlled trials

**DOI:** 10.1002/joa3.12698

**Published:** 2022-03-17

**Authors:** Chanavuth Kanitsoraphan, Pattara Rattanawong, Chol Techorueangwiwat, Jakrin Kewcharoen, Raktham Mekritthikrai, Narut Prasitlumkum, Parthav Shah, Hicham El Masry

**Affiliations:** ^1^ University of Hawaii Internal Medicine Residency Program Honolulu Hawaii USA; ^2^ Department of Cardiovascular Medicine Mayo Clinic Phoenix Arizona USA; ^3^ Department of Medicine, Division of Cardiology Loma Linda University Medical Center Loma Linda California USA; ^4^ Division of Cardiology Cook County Hospital Cook Illinois USA; ^5^ Department of cardiology University of California Riverside Riverside California USA

## Abstract

**Background:**

Posterior wall isolation (PWI) is an emerging approach in atrial fibrillation (AF) ablation, yet its efficacy remains controversial. This is the first meta‐analysis of randomized controlled trials (RCT) to evaluate the efficacy of PWI in AF ablation.

**Objective:**

To assess the efficacy of PWI in reducing atrial arrhythmia recurrence following initial AF ablation at long‐term follow‐ups when compared to conventional methods.

**Methods:**

We conducted a literature search from inception through September 2021 in EMBASE and MEDLINE databases. We included RCTs that compared outcomes in PWI and conventional approaches of AF ablation. Data from each study were combined using the random‐effects, generic inverse variance method of DerSimonian and Laird to calculate odds ratio (OR), and 95% confidence interval (CI).

**Results:**

Eight RCT from 2009 to 2020, including 1024 AF patients, were included. PWI did not decrease overall atrial arrhythmias recurrence (RR 0.96, 95% CI:0.88–1.05, *I* ^2^ = 31.6%, p‐value 0.393). However, the pooled analysis showed a significant decrease in AF recurrence in PWI compared to controlled approaches (RR 0.88, 95% CI:0.81–0.96, *I* ^2^ = 48.2%, *p*‐value .004). In the subgroup analysis, PWI significantly decreased AF recurrence in the studies that included only persistent AF (RR = 0.89, 95% CI:0.80–0.98, *I* ^2^ = 65.2%, *p*‐value .014). PWI significantly decreased AF recurrence when compared to PVI with roof line (RR 0.84, 95% CI 0.74–0.95, *I* ^2^ 0.00%, *p*‐value .008).

**Conclusion:**

Our study suggests that adding PWI significantly decreased AF recurrence in patients with persistent AF compared to controlled approaches. It highlights the importance of considering PWI during the initial procedure in this patient population.

## 1 INTRODUCTION

AF ablation is an established treatment in patients with symptomatic atrial fibrillation (AF). Atrial fibrillation ablation, when compared to medication therapy, has been shown effective in reducing AF recurrence, symptomatic AF, and AF burden.^1^, ^2^, ^3^, ^4^ The 2017 HRS/EHRA/ECAS/APHRS/SOLAECE expert consensus statement recommends catheter ablation for patients with symptomatic AF that is refractory or intolerant to at least one class I or III antiarrhythmic medication (Class I recommendation for paroxysmal AF [PAF], class IIa for persistent AF [PeAF], and class IIb for long‐standing PeAF).^5^ Pulmonary vein isolation (PVI) has been a standard approach for AF ablation. Additional ablation targets have been proposed to improve outcomes; however, there has been no class I recommendations for routine additional ablation in conjunction with PVI.^5^ Posterior wall isolation (PWI) is a promising emerging technique to improve AF ablation efficacy, particularly in patients who tend to have higher recurrence rates. However, the technique has faced challenges regarding its efficacy and safety since previous studies showed inconsistent data.^6^ The current guideline suggested PWI might be considered in initial or repeat ablation in PeAF or long‐standing PeAF (Class IIb).^5^ The STAR AF II trial suggested no benefits of additional lines performed beyond PVI in patients with PeAF.^7^ As more evidence on the efficacy of PWI emerges, this is the first meta‐analysis of RCT to evaluate the efficacy of PWI in AF ablation.

## 2 METHODS

### 2.1 Search strategy

The professional librarian performed the literature search from the EMBASE, PubMed, Scopus, Web of Science, and Cochrane databases from inception to September 2021 using a search strategy that includes the term “atrial fibrillation,” “pulmonary vein isolation,” “posterior wall isolation,” and “box isolation”. We manually reviewed references from review articles and systematic reviews for additional studies. Only full articles in English and studies conducted in RCTs were included. The quality assessment and bias risk assessment of the selected studies were conducted.

### 2.2 Inclusion and exclusion criteria

The eligibility criteria included the following.

(1) Randomized control trial studies reporting endpoints of atrial fibrillation or atrial arrhythmia recurrence after AF ablation with or without PWI.

(2) Adjusted or unadjusted RR with 95% confidence interval, or adequate raw data for calculation were provided.

Study eligibility was independently determined by two investigators (CK and PR), and differences were resolved by mutual consensus. Studies with overlap or duplicated populations were excluded.

### 2.3 Data extraction

The included studies were reviewed for the type of study, country of origin, type of AF, total population, mean age, PWI techniques and techniques in control arms, mean follow‐up, the definition of recurrence, and conclusion. Extracted data were collected using standardized forms. Overall atrial arrhythmia was defined as the combination of AF, atrial flutter, or atrial tachycardia.

### 2.4 Statistical analysis

We performed a meta‐analysis of the included studies using a fixed‐effect model. We pooled the point estimates of RR from each study using the generic inverse‐variance method of Der Simonian and Laird. Heterogeneity was quantified using the *I* ^2^ statistics, which range from 0 to 100% and *I* ^2^ > 50% indicates substantial heterogeneity. Publication bias was assessed using a funnel plot, and Egger's regression test with a *p*‐value of <.05 was considered significant. All statistical analyses were performed using Stata version 14.2 TX: StataCorp; 2015.

## 3 RESULTS

### 3.1 Search results

**FIGURE 1:**
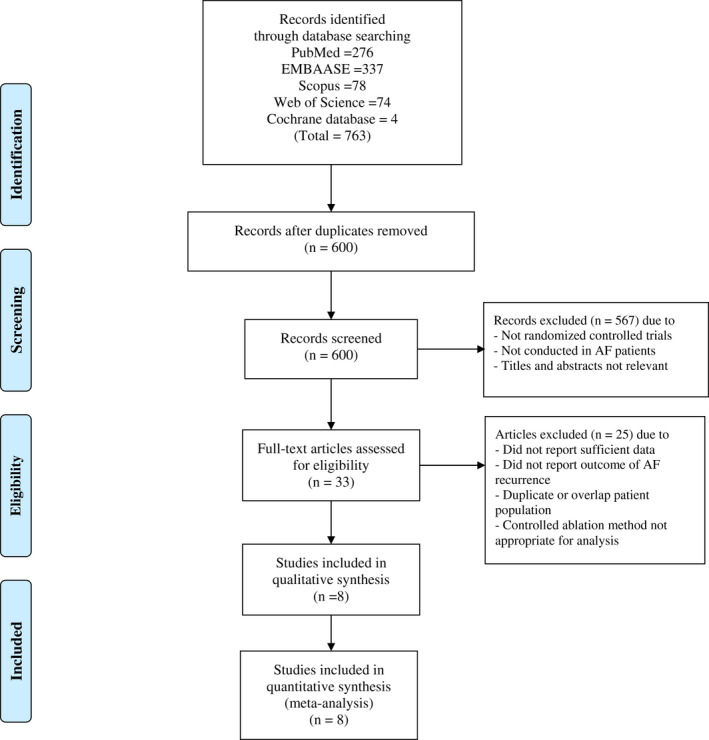
Search methodology and selection process

Our search strategy yielded 763 potentially relevant articles (331 articles from EMBASE, 276 articles from PubMed, 78 from Scopus, 74 from Web of Science, and 4 from the Cochrane database). After the exclusion of duplicated articles, 600 articles underwent title and abstract review. At this stage, 567 articles were excluded as they were not randomized controlled trials, were not conducted in AF patients, or the titles and abstracts were not relevant. This left 33 articles for full‐length review. A further 25 studies were excluded as they were overlap or duplicated patients' populations or did not report outcomes and did not provide sufficient data to calculate RR. Therefore, a total of 8 studies were included in this meta‐analysis. Figure 1 outlines the search and literature review process.

### 3.2 Descriptions of included studies

Eight studies with a total of 1024 AF patients undergoing initial AF ablation were analyzed and included in this meta‐analysis.^8^, ^9^, ^10^, ^11^, ^12^, ^13^, ^14^, ^15^, ^16^ Of these patients, 512 (50.0%) underwent PWI and 512 (50.0%) underwent controlled approaches. One hundred and seventy‐eight (34.8%) undergoing PWI and 189 patients (36.9%) undergoing controlled approaches had atrial arrhythmia recurrence at follow‐up. The characteristics of included studies are outlined in Table 1.

**TABLE 1:** Summarized characteristics of individual included studies

First author, year	Country	Institutions	Type of AF	N	Posterior Wall isolation ablation technique	Control	Mean follow up (months)	Recurrence definition	Complications	Conclusion
Aryana, 2020	USA Japan	Japan Red Cross Yokohama‐city Bay Hospital and Mercy General Hospital and Dignity Health Heart and Vascular Institute	PeAF/LSPe AF	110	CPVI (cryoballoon) + PWI (cryoballoon ablation of bounded by LA roof, left pulmonary vein, right pulmonary vein, and posterior inferior border) + CTI 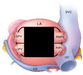	CPVI (cryoballoon) + CTI 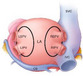	12	AF,AT, atrial flutter >30 seconds Method: ECG at each follow‐up visit, 7‐day to 14‐day mobile cardiac telemetry monitoring at 3, 6, and 12 months post‐ablation, unless a cardiac implantable electronic device existed.	PWI: 1 persistent phrenic nerve palsy, 1 bradycardia requiring pacemaker, 1 groin vascular complication Control: 1 Heart failure exacerbation, 1 Pericarditis, 1 Pericardial effusion	PVI + PWI using cryoballoon is associated with a significant reduction in atrial fibrillation recurrence
Chilukuri, 2011	USA	The John Hopkins Hospital	PAF 79%, PeAF 21%	30	Single ring (box) isolation: single continuous lesions at the anterior aspect of PV joined with a roof line superiorly and a floor line inferiorly 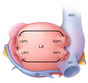	CPVI (without interpulmonary isthmus line) 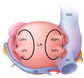	10 ± 2	AF, AT, atrial flutter >30 seconds Method: Daily 30‐second measurement of heart rhythm with the portable ECG monitoring device	PWI: 1 embolic stroke, 1 cardiac tamponade, 1 femoral arterial pseudoaneurysm, 1 abdominal wall hematoma Control: None	The efficacy of box isolation is similar to circumferential PVI protocol for AF ablation.
JS Kim, 2014	Korea	Korea University Guro Hospital	PeAF	120	CPVI (without interpulmonary isthmus line) + POBI (linear ablation along the roof and posterior inferior wall) + anterior wall of LA + CTI 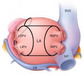	CPVI (without interpulmonary isthmus line) + LA linear ablation on the roof + anterior wall of LA + CTI 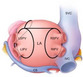	12	AF or atrial flutter Method: ECG at every visit, 48‐hour Holter monitoring at 1, 3, 6, and 12 months.	PWI: None Control: None	Additional POBI after anterior wall linear lesions and PVI can reduce AF recurrence in PeAF
Lee, 2019	Korea	Kyung Hee University Medical College, Korea University Cardiovascular Center, Ewha Womans University, Yonsei University Health System, and Hanyang University	PeAF	217	CPVI + POBI (linear ablation along the roof and posterior inferior wall) + CTI 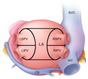	CPVI + CTI 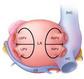	16.2 ± 8.8	AF or AT >30 seconds Method: ECG at every visit and 24‐hour Holter at 3 and 6 months and then every 6 months thereafter	PWI: 4 cardiac tamponades, 2 sinus node dysfunction, 1 atrioesophageal fistula, 3 pericarditis, 2 pseudoaneurysm Control: 4 cardiac tamponade, 1 SA node dysfunction, 1 atrioesophageal fistula, 1 pericarditis	In patients with PeAF, an empirical POBI did not improve the rhythm outcome of the catheter ablation
Lim, 2012	Australia, Singapore	Westmead Hospital (Australia), National University Hospital (Singapore), Liverpool hospital and University of New South Wales (Australia)	PAF, PeAF or long‐standing AF	220	*Single‐ring (box) isolation*: single continuous lesions at the anterior aspect of PV joined with a roof line superiorly and a floor line inferiorly + CTI 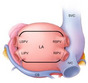	*Wide antral isolation*: CPVI (without interpulmonary isthmus line) + LA linear ablation on the roof + CTI 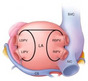	24	AF,AT, atrial flutter >30 seconds Method: ECG or 7‐day Holter at 6 and 12 months.	PWI: 1 cardiac tamponade, 2 ischemic stroke Control: 1 cardiac tamponade, 1 ischemic stroke	Single‐ring isolation resulted in fewer AF recurrences than wide antral isolation, although organized AT and overall atrial arrhythmia recurrences were similar.
Mun, 2012	Korea	Yonsei University Health System	PAF	156	CPVI + POBI (linear ablation along the roof and posterior inferior wall) 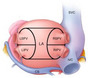	CPVI 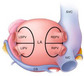	15.6 ± 5.0	AF or AT >30 seconds Method: ECG at every visit. 24‐hour/ 48‐hour and/or event recorder at 3, 6, and 12 months.	PWI: 3 pericarditis Control: 1 pericardial effusion, 1 percarditis	Additional linear POBI ablations to CVPI did not improve clinical outcome.
Pak, 2020 (PEACEFUL)	Korea	Yonsei University Health System, Korea University Cardiovascular Center, and Ewha Womans University	PeAF who converted to PAF by AAD	114	CPVI + POBI (linear ablation along the roof and posterior inferior wall) + CTI (posterior inferior linear ablation) 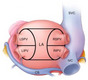	CPVI + CTI 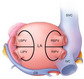	23.8 ± 10.2	AF or AT >30 seconds Method: ECG at every visit, 24‐hour Holder at 3 and 6 months and then every 6 months	PWI: 1 phrenic nerve palsy Control: 1 femoral AV fistula, 1 hemopericardium, 1 left inferior pulmonar vein stenosis	The addition of POBI to CVPI did not improve the outcome in patients with PeAF who previously converted to PAF by AAD.
Tamborero, 2009	Spain	University of Barcelona Hospital Clinic and Universitari de Barcelona Hospital Clinic	PAF, PeAF or long‐standing AF	120	CPVI + POBI (linear ablation along the roof and posterior inferior wall) 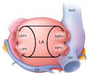	CPVI (without interpulmonary isthmus line) + LA linear ablation on the roof 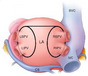	9.8 ± 4.3	AF or atrial flutter Method: 48‐hour Holter monitoring before visits at 1, 4 and 7 months, then every 6 months.	PWI: 1 transient cerebrovascular ischemia, 1 transient inferior myocardial ischemia Control: 2 transient cerebrovascular ischemia, 1 transient inferior myocardial ischemia	Isolation of the left atrial posterior wall did not offer additional benefit over a single roof line lesion after CPVI.

Abbreviations: AAD, antiarrhythmic drugs; AF, atrial fibrillation; AT, atrial tachycardia; CS, coronary sinus; CVPI, circumferential pulmonary vein isolation; IVC, inferior vena cava; POBI, posterior box isolation; CTI, cavotricuspid isthmus; LA, left atrium; LIPV, left inferior pulmonary vein; LSPe AF, Long‐standing persistent AF; LSPV, left superior pulmonary vein; PeAF, Persistent AF; PAF, Paroxysmal AF; PWI, posterior wall isolation; RIPV, right inferior pulmonary vein; RSPV; right superior pulmonary vein; SVC, superior vena cava.

### 3.3 Overall meta‐analysis results

**FIGURE 2:**
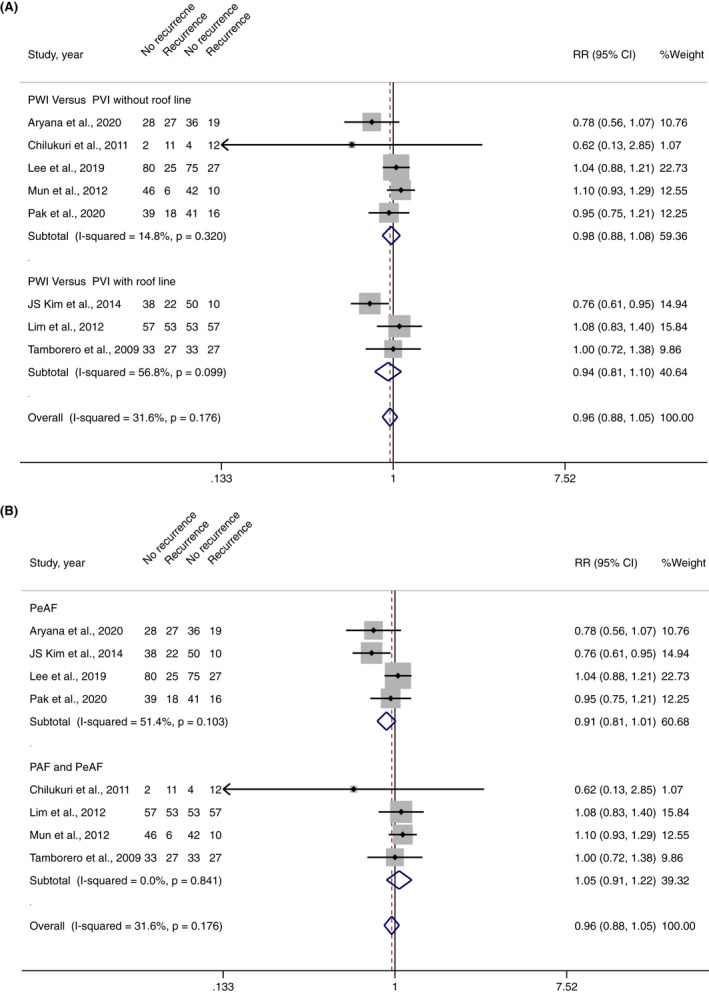
(A) Forest plot of PWI and overall atrial arrhythmia recurrences stratified by a subgroup of PWI versus PVI without roof line and PWI versus PVI with roof line. (B) Forest plot of PWI and overall atrial arrhythmia recurrences stratified by subgroup of PeAF and PeAF with PAF

**FIGURE 3:**
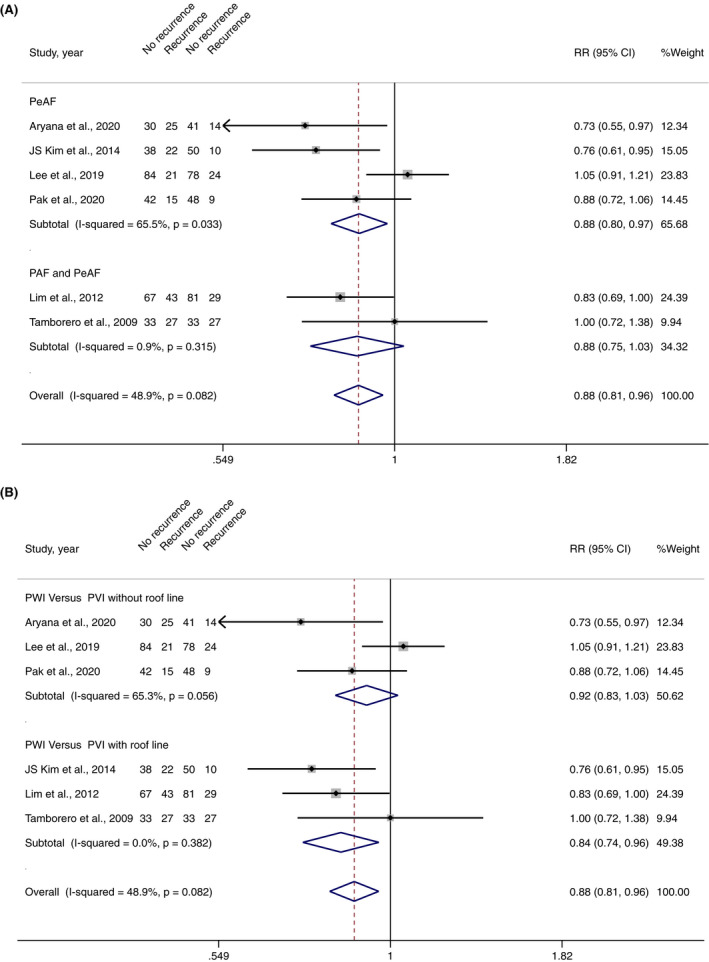
(A) Forest plot of PWI and AF recurrences stratified by the subgroup of PWI versus PVI without roof line and PWI versus PVI with roof line; (B) Forest plot of PWI and AF recurrences stratified by a subgroup of PeAF and PeAF with PAF

In the overall pooled analysis of 8 studies, PWI was not significantly associated with a decrease in overall atrial arrhythmia recurrences (RR 0.96, 95% CI 0.88–1.05, *I* ^2^ = 31.6%, *p*‐value .393) (Figure 2). However, 6 of 8 studies^8^, ^10^, ^12^, ^13^, ^15^, ^16^ reported AF recurrence as an outcome, in which pooled analysis showed that PWI significantly decreased the risk of AF recurrence (RR 0.88, 95% CI 0.81–0.96, I^2^ 48.4%, *p*‐value .003) (Figure 3).

### 3.4 Outcomes by type of AF

#### 3.4.1 Persistent AF

Four studies included a total of 547 PeAF patients undergoing initial AF ablation (273 PWI and 274 controlled approaches).^8^, ^10^, ^12^, ^16^ AF recurred in 59 patients (21.5%) undergoing PWI and 84 patients (30.3%) undergoing controlled approaches. Atrial arrhythmias recurred in 72 patients (26.3%) undergoing PWI and 92 patients (33.2%) undergoing a controlled approach. Pooled analysis demonstrated that PWI significantly decrease AF recurrences (RR 0.88, 95% CI 0.80–0.97, *I* ^2^ 65.5%, *p*‐value .019) (Figure 3B) but did not decrease overall atrial arrhythmia recurrences (RR 0.91, 95% CI 0.81–1.01, *I* ^2^ 51.4%, *p*‐value .073) in PeAF population (Figure 2B).

#### 3.4.2 Paroxysmal and persistent AF

Four studies involved 473 non‐specific AF patients (included both PeAF and PAF) undergoing initial AF ablation (238 PWI and 235 controlled approaches).^9^, ^13^, ^14^, ^15^ Atrial arrhythmias recurred in 106 patients (39.6%) undergoing PWI and 97 patients (36.1%) undergoing the conventional approach. Pooled analysis demonstrated that PWI did not decrease AF recurrences (RR 0.87, 95% CI 0.75–1.02, *I* ^2^ 0.00%, *p*‐value .090) (Figure 3B) as well as overall atrial arrhythmia recurrences (RR 1.05, 95% CI 0.91–1.22, *I* ^2^ 0.0%, *p*‐value .515) (Figure 2B) in the non‐specific population of AF.

### 3.5 Outcomes by ablation approaches

#### 3.5.1 PWI versus PVI without roof line

Five studies including a total of 564 patients undergoing initial AF ablation (282 PWI and 282 PVI without roof line) reported overall atrial arrhythmia recurrences.^8^, ^9^, ^12^, ^14^, ^16^ Three of which reported AF recurrences.^8^, ^12^, ^16^ Pooled analysis demonstrated that PWI did not decrease overall atrial arrhythmia recurrences (RR 0.98, 95% CI 0.88–1.08, *I* ^2^ 14.8%, *p*‐value .657) (Figure 2A) or AF recurrences (RR 0.92, 95% CI 0.83–1.03, *I* ^2^ 65.3%, *p*‐value .138) (Figure 2B).

#### 3.5.2 PWI versus PVI with roof line

Three studies including a total of 460 patients undergoing initial AF ablation (230 PWI and 230 PVI with roof line) reported overall atrial arrhythmia and AF recurrences.^10^, ^13^, ^15^ Pooled analysis demonstrated that PWI significantly decreased AF recurrences (RR 0.84, 95% CI 0.74–0.95, *I* ^2^ 0.0%, *p*‐value .008) (Figure 3A), but did not decrease overall atrial arrhythmia recurrences (RR 0.94, 95% CI 0.81–1.10, *I* ^2^ 56.8%, *p*‐value .445) (Figure 2A) when compared to PVI with roof line.

#### 3.5.3 Complications

**FIGURE 4:**
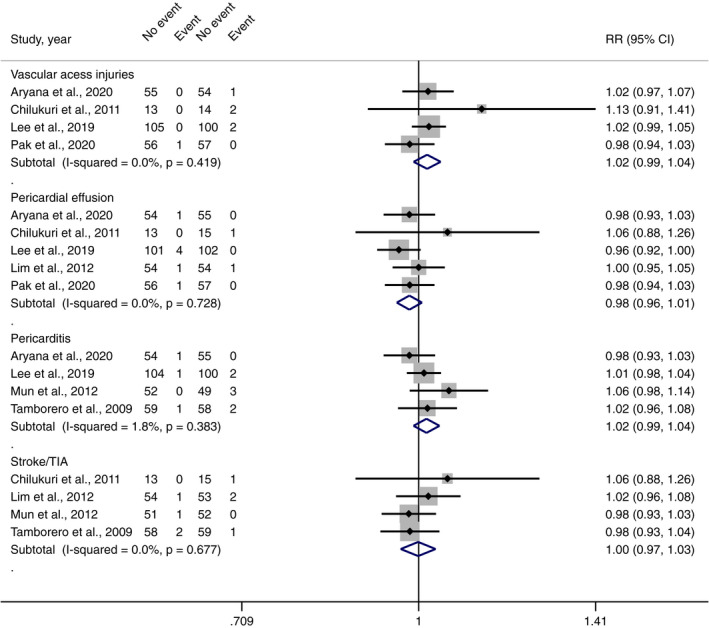
Forest plot of the risk of complications from PWI compared with controlled approaches

There was no significant difference risk of vascular access (RR 1.02, 95% CI 0.99–1.04, *I* ^2^ 0.0%, *p*‐value .222), pericardial effusion (RR 0.98, 95% CI 0.96–1.01, *I* ^2^ 0.0%, *p*‐value .147), pericarditis (RR 1.02, 95% CI 0.99–1.04, *I* ^2^ 1.8%, *p*‐value .240), and stroke or TIA (RR 1.00, 95% CI 0.97–1.03, *I* ^2^ 0.0%, *p*‐value .950) between PWI and controlled approaches (Figure 4).

#### 3.5.4 Sensitivity analysis

We conducted a sensitivity analysis by excluding one study at a time to assess the stability of the results of the meta‐analysis. None of the results were significantly altered in the overall analysis.

### 3.6 Publication and risk study bias

**FIGURE 5:**
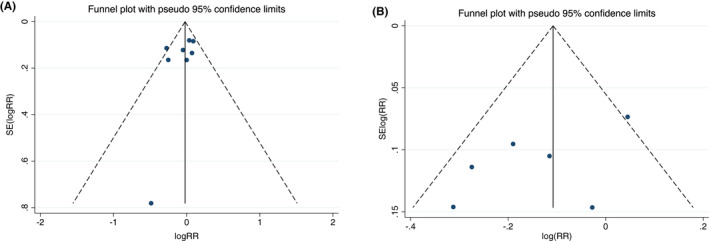
Funnel plot of (A) PWI and overall atrial arrhythmia recurrences; B) PWI and AF recurrences

**FIGURE 6:**
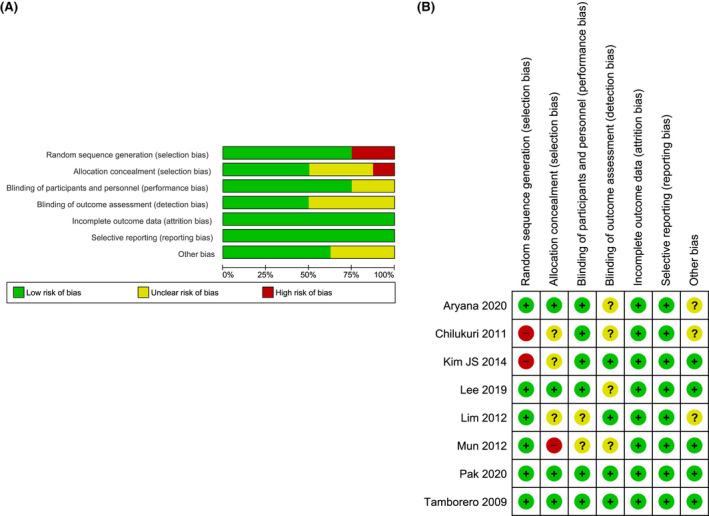
The quality assessment and bias risk assessment of the selected studies; (A) risk of bias graph; (B) risk of bias summary

We examined the contour‐enhanced funnel plot of the included studies to investigate potential publication bias. No significant publication bias was observed on the funnel plot of overall atrial arrhythmias and AF; however, in the limitation of a small number of included study. (Figure 5A and Figure 5B, respectively). Meanwhile, there was no small study bias observed in the Egger's test on overall atrial arrhythmias and AF analysis (*p* = .192 and *p* = .174, respectively). The quality assessment and bias risk assessment of the selected studies are shown in Figure 6.

## 4 DISCUSSION

We conducted a meta‐analysis of RCTs evaluating the addition of PWI to conventional ablation. The main finding from this meta‐analysis is that the addition of PWI did not significantly improve efficacy in reducing arrhythmia recurrence.

In PAF, catheter ablation with radiofrequency or cryotherapy is known to be superior in maintaining sinus rhythm compared to antiarrhythmic therapy.^2^, ^3^, ^4^, ^17^ Nonetheless, therapy to maintain sinus rhythm in PeAF is less effective, and this reflects in weaker recommendations in the current guideline.^5^ The addition of left atrial roof line ablation was used by some investigators in an effort to improve clinical success, however increased risk of atrial flutter was noted on follow up (reference 14,18,19) An analysis of AF recurrence from the catheter ablation versus antiarrhythmic drug therapy in atrial fibrillation (CABANA) trial suggested significantly reducing recurrence of any AF by 48% and symptomatic AF by 51%, when compared to drug therapy over 5 years of follow up.^1^ Although, the trial stipulates that all catheter ablation involved PVI, with additional ablation at the investigator's discretion. Whether additional PWI would benefit AF recurrence remains controversial.

The posterior left atrial wall has a critical role in the initiation and maintenance of AF. Several anatomical and electrophysiologic properties increase the arrhythmogenicity of the posterior wall. Both the pulmonary veins and the posterior wall are embryologically derived from the same tissue (mediastinal myocardium). The posterior wall and PV also have shorter action potential durations and slower phase 0 upstroke velocity.^20^ The myocardial fibers’ orientation in the PV antra and posterior wall is distinct, allowing reentry from anisotropic conduction. Ganglionic plexi are most prevalent in the posterior wall of the left atrium.^21^ The posterior wall may be disproportionately affected by the pressure stress, and that has been correlated with low voltage and electrical scar.^22^, ^23^, ^24^ These areas of low voltage and electrical scar are predictive of poor outcomes after catheter ablation in persistent AF. The posterior wall has emerged as an additional target of ablation, especially in a patient undergoing ablation for persistent atrial fibrillation.

Our study suggested that the addition of PWI significantly decreased AF recurrence in PeAF but failed to decrease overall atrial arrhythmia recurrence. A study by Lim et al.^13^ demonstrated that PWI increased the incidence of reentrant tachycardia, which is likely attributable to posterior wall reconnection or even persistent epicardial connection (possibly due to the inability to achieve transmural ablations). We suspect that the decrease in AF recurrence is offset by an increase in the risk of reentrant flutter. Larger randomized controlled trials are needed to elucidate the magnitude of this phenomenon. Importantly, our study showed that the addition of PWI was not associated with increased acute/short‐term complications. Whether increased thermal injury to the esophagus and risk of atrio‐esophageal fistula is associated with the addition PWI remains unclear: this is related to the rare occurrence of this complication but invites caution.

### 4.1 Different criteria/techniques for posterior wall isolation

There is slight heterogeneity in PWI techniques among studies included in this meta‐analysis, as outlined in Table 1. The key methods for PWI include a single ring^25^, ^26^, ^27^ and PVI plus box lesion set.^28^, ^29^, ^30^ There has also been emerging use of cryoablation with more recent generations, of which the efficacy compared to radiofrequency (RF) ablation remains controversial.^31^, ^32^ More data would be needed to elucidate the impact of cryoablation versus RF ablation on the finding from this meta‐analysis.

### 4.2 Comparison with other meta‐analyses

A recent meta‐analysis by Thiyagaragjah et al., which includes 17 studies, evaluated the efficacy and safety of PWI during AF ablation.^33^ The study concluded that PWI could be achieved in a large portion of cases with satisfactory 12‐month freedom of atrial arrhythmia. Of note, there were only three RCT comparing PWI with PVI directly, and the interpretation of combined conflicting data would be limited. Studies with cryoablation were excluded from the analysis.

### 4.3 Limitations

Our study has a few limitations. There was substantial heterogeneity among studies. Sensitivity analysis was undertaken. There is slight variation in techniques performed, patient population, and definitions of outcomes as outlined in Table 1. The number of participants included in the trial is relatively small and may lead to underpower in subgroup analyses. Moreover, there was a limitation of funnel plot interpretation from a small number of included studies.

## 5 CONCLUSIONS

Our study showed that the addition of PWI to routine PVI in AF ablation significantly decreased AF recurrences after AF ablation, particularly in patients with PeAF. There were no significant differences in overall atrial arrhythmia recurrences with the addition of PWI to routine PVI, highlighting the increased risk of reentrant arrhythmias after this intervention.
